# Circ-06958 Is Involved in Meat Quality by Regulating Cell Proliferation Through miR-31-5p/AK4 Axis in Pigs

**DOI:** 10.3390/cells14181416

**Published:** 2025-09-10

**Authors:** Xiaohan Zhang, Rongru Zhu, Xiaoxu Wu, Minghang Chang, Yuanlu Sun, Liang Wang, Ming Tian, Dongjie Zhang, Di Liu, Xiuqin Yang

**Affiliations:** 1 College of Animal Science and Technology, Northeast Agricultural University, Harbin 150030, China; zhangxiaohan21sdk@163.com (X.Z.); zhurongruzi@163.com (R.Z.); wxiaox2022@163.com (X.W.); neauchangminghang@163.com (M.C.); sunyuanlu2023@126.com (Y.S.); 2 Institute of Animal Husbandry, Heilongjiang Academy of Agricultural Sciences, Harbin 150086, China; wlwl448@163.com (L.W.); tianming@haas.cn (M.T.); djzhang8109@haas.cn (D.Z.)

**Keywords:** circRNA, miR-31-5p, porcine skeletal muscle satellite cells, proliferation, *AK4*, meat quality

## Abstract

Circular RNA (CircRNA) can regulate gene expression through acting as a competitive endogenous RNA (ceRNA), thus becoming involved in various biological processes. However, little was known about the role of circRNA in the formation of meat quality in pigs. Here, circRNAs were first characterized in muscles with differential meat quality and myofiber composition, longissimus thoracis, and semitendinosus muscles, with RNA-sequencing (RNA-seq). A total of 1126 differentially expressed circRNAs were identified. Among them, Circ-06958 is highly expressed in both muscles. Circ-06958 originated from Long-chain acyl-CoA synthetase 1 (*ACSL1*), a gene involved in muscle development. Circ-06958 was then characterized experimentally for the first time. Next, it was revealed that Circ-06958 increased proliferation of muscle cells, including porcine skeletal muscle satellite cells (PMSCs) and C2C12 myoblasts, by promoting cell cycle progression. Circ-06958 was mainly localized in cytoplasm, indicating it can function as a ceRNA. A regulatory axis Circ-06958/miR-31-5p/Adenylate Kinase 4 (*AK4*) axis was constructed with molecular biology techniques. Afterward, it was shown that miR-31-5p inhibited cell proliferation by affecting cell cycle progression in the two cells, while *AK4* increased it. We made it clear that Circ-06958 promoted muscle cell proliferation via the miR-31-5p/*AK4* axis. The results will contribute to further revealing the mechanisms through which meat quality generates.

## 1. Introduction

Pork is the main source of meat for the Chinese people. Its consumption accounts for more than half of the total meat consumption in the Chinese diet. With the improvement of living standards, consumers are paying more attention to meat quality. Meat quality is a complex trait composed of many physicochemical properties including pH, tenderness, flavor, meat color, water holding capacity, and marbling [1]. It was revealed that muscle fiber composition is associated with meat color, pH, and water-holding capacity [2,3,4,5,6]. Additionally, muscle development is also an important factor associated with meat quality [7].

Meat quality is determined by a combination of genetic and environmental factors. It is difficult to improve meat quality with conventional cross breeding, feeding, and management, etc. The development of molecular biology techniques provides useful tools for characterizing the hereditary basis of meat quality. Some genetic factors have been involved in meat quality, including protein-encoding genes, noncoding RNA (ncRNA), and epigenetic modification as well. Lnc-ADAMTS9 plays an essential role in myogenesis through the ERK signaling pathway [8]. Lnc-MEG8 antagonizes the inhibitory effect of miR-22-3p on key genes of muscle formation, promotes myotube formation, and plays a key role in yak muscle development [9]. *ACTC1*, *ACTG2*, and *ACTN2* were associated with fast and slow muscle fiber conversion [10]. *LRP1* was revealed to regulate muscle fiber development together with genes involved in myoblast proliferation and apoptosis, thereby affecting meat quality in chickens [11]. However, research on the role of circular RNA (circRNA) in meat quality is limited.

CircRNA is a special type of ncRNA with a closed-loop structure. Compared with linear RNA, circRNA has no 5′-cap and 3′ poly(A) tail in structure, and less sensitivity to nuclease. CircRNA can impair premature mRNA processing and ribosome biogenesis by interacting with proteins [12]. Those nuclear-retained circRNAs were found to reduce the transcription of their parent genes [13]. CircRNAs also can act as competitive endogenous RNAs (ceRNAs) to absorb microRNAs (miRNAs), thereby regulating the expression of target genes [14,15]. Currently, it has been shown that CircRRAS2 promotes the differentiation of bovine muscle stem cells into myotubes [16]. circMYLK4 was identified to be a regulator of fast/slow myofiber conversion [17]. It was also confirmed that circRNAs had a role in determining meat quality through performing RNA-sequencing (RNA-seq) on muscles with differential meat quality in pigs [18,19,20].

However, this is far from revealing the specific role and underlying mechanisms of circRNA in meat quality. This study was designed to characterize circRNAs associated with differential meat quality and myofiber composition between the longissimus thoracis and semitendinosus based on RNA-seq. The sequences and effects of candidate circRNA on proliferation of muscle cells were then characterized, and a regulatory ceRNA axis was constructed. The results will contribute to revealing the mechanisms through which meat quality generates, and thereafter contribute to controlling meat quality, which will better satisfy consumers.

## 2. Materials and Methods

### 2.1. Nucleic Acid Isolation, RNase R Digestion, cDNA Synthesis, and PCR

Min pigs were used here. The pigs were slaughtered strictly according to the Chinese national standard for pig slaughtering (GB/T 17236–2019). Briefly, the individuals were electrically stunned, followed by exsanguination. Muscles were collected immediately after the pigs were slaughtered. Genomic DNA (gDNA) was isolated with normal phenol-chloroform method. That is, the muscle sample was first digested with Proteinase K and SDS, and extracted with phenol-chloroform isopropanol by centrifuge at 12,000× *g*. The precipitates were washed with 75% ethanol and dissolved with ddH_2_O. Total RNA was isolated using TRIzol reagent (Invitrogen, Carlsbad, CA, USA). Briefly, 50–100 mg muscle tissues were ground in liquid nitrogen, mixed with 1 mL TRIzol reagent, and incubated for 5 min to dissociate the nucleoprotein complex completely. A total of 0.2 mL chloroform was added, and the mixture was incubated for another 2–3 min and centrifuged for 15 min at 12,000× *g* at 4 °C. The upper aqueous phase was transferred to a new tube and treated with isopropanol and 75% ethanol in sequential order. The precipitate was dissolved in RNA-free water, and the integrity, quality, and quantity of RNA was measured. If needed, total RNA was digested with 3 U/µg RNase R (Beyotime, Shanghai, China) for 15 min at 37 °C. Cytoplasmic and Nuclear RNA was obtained using Cytoplasmic and Nuclear RNA Purification Kit (Norgen Biotek, Thorold, ON, Canada). Reverse transcription (RT) was performed with the HiScript III 1st Strand cDNA Synthesis Kit (+gDNA wiper) (Vazyme, Nanjing, China). PCR was performed with 2 × Taq PCR Master Mix (Vazyme) according to the protocol of the manufacturer. The reaction conditions were as follows: pre-denaturation at 94 °C for 5 min, denaturation at 94 °C for 30 s, annealing at 55~60 °C for 30 s, extension at 72 °C for 1 min, 32 cycles, final extension at 72 °C for 7 min, and stored at 16 °C. Real-time quantitative PCR (qPCR) was conducted in a total volume of 20 μL containing 1 μL cDNA, 0.4 μL of each forward and reverse primer (10 μmol/L), and 10 μL 2 × ChamQ Universal SYBR qPCR Master Mix (Vazyme), each with triplicate. The reaction was performed on the QuantStudio 3 Real-Time PCR System (ThermoFisher Scientific, Massachusetts, MA, USA) with the following cycle settings: 30 s at 95 °C, followed by 40 cycles of 5 s at 95 °C and 20 s at 60 °C. The data was analyzed with 2−ΔΔCt method. β-actin was selected as reference for quantification of mRNA and circRNA, while U6 was used for miRNA quantification. All primers were designed with primer premier 5.0 and synthesized by Genesoul Technology (Harbin, China). The primer sequences were listed in Table S1-A.

### 2.2. CircRNAs Characterization

We have obtained transcriptome landscape of miRNA, lncRNA, and mRNA in longissimus thoracis and semitendinosus muscles of Min pigs with RNA-seq (CRA020799; https://ngdc.cncb.ac.cn/gsa (accessed on 17 April 2023)) [21]. Based on this, the circRNAs were analyzed. DESeq2 package (v3.20)was used for screening differentially expressed (DE) circRNAs with criteria *p <* 0.05 and absolute log2fold-change ≥ 1. Functional enrichment was performed with the Gene Ontology (GO) and Kyoto Encyclopedia of Genes and Genomes (KEGG) resources. The protein–protein interaction (PPI) was established using the STRING (the Retrieval of InteractingGenes/Proteins) database and Cytoscape software (v3.7.1).

Based on sequences obtained by RNA-seq, divergent primers across the Back-Splice Site and ordinary convergent primers were designed to characterize Circ-06958 experimentally. Using the two primer pairs, PCR was performed with gDNA and cDNA template, respectively. The products were inserted into pMD-18T vector (Takara, Dalian, China), and sequenced by Genesoul Biol (Harbin, China).

### 2.3. Plasmids and Oligonucleotide Sequences

The full-length sequence of Circ-06958 and the complete coding sequences (CDS) of AK4 were amplified with RT-PCR from cDNA obtained from muscles, respectively, to construct overexpression plasmids. Circ-06958 sequences were inserted into pCD2.1-CIR at enzyme sites Kpn I and Bam HI. The CDS of *AK4* was inserted into pCMV-HA at the enzyme sites EcoR I and Kpn I. At the same time 3′-UTR of AK4 gene was amplified from gDNA. Luciferase reporter genes containing the full-length sequence of circ-06958 and 3’-UTR of *AK4*, respectively, were constructed with psiCHECK-2 backbone at Kpn I and Hind III restriction sites. Site-directed mutagenesis was performed with overlapping extension PCR, as described previously, to obtain mutant-type reporter genes [22]. Primers used for plasmid construction were listed in Table S1-B. MiRNA mimics, inhibitor, and siRNAs against mouse and pig *AK4* gene, respectively, were synthesized by General Biol (Hefei, China), and the sequences are listed in Table S1-C.

### 2.4. Cell Culture and Transfection

C2C12 (CRL-1772) and PK15 (CCL-33) cell lines (Genetimes, Shanghai, China) were cultured in Dulbecco’s Modified Eagle’s Medium (DMEM)/High Glucose (SEVEN, Beijing, China) containing 10% fetal bovine serum (FBS; OPCEL, Inner Mongolia, China) and 1% penicillin-streptomycin (Invitrogen). The cells were cultured at 37 °C with 5% CO_2_ and the medium was changed every 48 h. Porcine skeletal muscle satellite cells (PMSCs; MINGZHOUBIO, Ningbo, China) were cultured in DMEM/Nutrient Mixture F-12 (F12) containing 10% FBS (Gibco, Carlsbad, CA, USA), 1% penicillin-streptomycin, and 1% growth factor (MINGZHOUBIO). The plasmid was transiently transfected into cells using Lipofectamine 2000 reagent (Invitrogen), and the ratio of plasmid to reagent was 1:2.5.

### 2.5. Cell Counting Kit 8 Assay

Cells were inoculated at a density of 2500 for 16 h in 96-well plates. Then, we transfected the overexpression plasmids, or oligonucleotides, into the cells for 24 h, which was designated as 0 h. 10 μL CCK-8 reagent (Cell Counting Kit 8, Beyotime) was added to each well at 0, 24, 48, 72, 96, and 120 h. The optical density (OD) at 450 nm was measured with the Tecan Microplate Reader Infinite F50 (Tean GENios, Mannendorf, Switzerland).

### 2.6. 5-Ethynyl-2′-Deoxyuridine Incorporation Assay

5-ethynyl-2′-deoxyuridine (EdU) staining was carried out with BeyoClick™ EdU-555 kit (Beyotime) as described previously [23]. Briefly, 2800 cells were seeded into a 96-well plate and cultured at 37 °C for 14 h. The cells were transfected with plasmids or oligonucleotides. At 24 h post-transfection, the cell medium was replaced with a medium containing 10 µM EdU and cultured for another 2 h. The cells were then incubated with Azide 555 for 30 min at room temperature after fixed and permeabilized. Finally, the cells were stained with Hoechst 33342 for 10 min and observed with the Olympus inverted fluorescence microscope IX71 (Olympus, Tokyo, Japan). The fluorescence was measured at 346 nm (excitation)/460 nm (emission).

### 2.7. Western Blotting

Cells were seeded in six-well plates and cultured until reaching approximately 50% confluence, then transfected with an overexpression vector or oligonucleotides. Forty-eight hours post-transfection, total protein was extracted using RIPA buffer (Beyotime) supplemented with a protease inhibitor (Invitrogen) and quantified using the enhanced BCA protein assay kit (Beyotime). A total of 25–30 µg of protein was separated by SDS-polyacrylamide gel electrophoresis and transferred to a polyvinylidene fluoride (PVDF) membrane (Millipore, Shanghai, China). The membrane was blocked with 5% skim milk and incubated with primary antibodies: anti-AK4 (1:500 dilution; Proteintech, Wuhan, China), anti-PCNA (1:5000 dilution; Proteintech), and anti-β-tubulin (1:20,000 dilution; Proteintech). β-tubulin was used as a loading control. Membranes were then probed with goat anti-mouse and donkey anti-rabbit secondary antibodies (1:20,000 dilution; LI-COR, Lincoln, NE, USA). Protein bands were detected using the Odyssey^®^ DLx (LI-COR) near-infrared imaging system (LI-COR), and the relative intensities of the bands were quantified using ImageStudio software (v 5.2.5).

### 2.8. Flow Cytometry Analysis

Cells were seeded in six-well plates at a density of 1.2 × 10^5^ per well. When they reached 50% confluence, the cells were transfected with plasmids or oligonucleotides for 24 h. The cells were washed with phosphate-buffered solution (PBS) twice, digested with trypsin for 1 min, and resuspended using high-glucose DMEM. Then, the cells were collected and stained with a cell cycle staining Kit (MultiSciences, Hangzhou, China). Briefly, 1 mL DNA-staining solution and 10 μL permeabilization solution were added into the cells, with vortex oscillation for 10 S to mix thoroughly. After being incubated for 30 min at room temperature, the cell cycle was analyzed with the Agilent NovoCyte Flow Cytometer (Palo Alto, Santa Clara, CA, USA).

### 2.9. Dual-Luciferase Reporter Gene Analysis

The reporter genes and mimics were co-transfected into PK15 cells. After 48 h post-transfection, the cells were collected, and luciferase activity was analyzed by using the dual-luciferase reporter gene assay kit (Beyotime). The relative luciferase activity was measured as a ratio of Renilla to firefly luciferase value.

### 2.10. Statistical Analysis

All experiments were performed three times, each with triplicates. Statistical analyses were conducted with GraphPad Prism (version 9.5.1; GraphPad, San Deigo, CA, USA), and an unpaired *t*-test was used to compare the differences between groups. *, *p <* 0.05; **, *p <* 0.01.

## 3. Results

### 3.1. Analysis of Differential circRNA Expression

**Figure 1:**
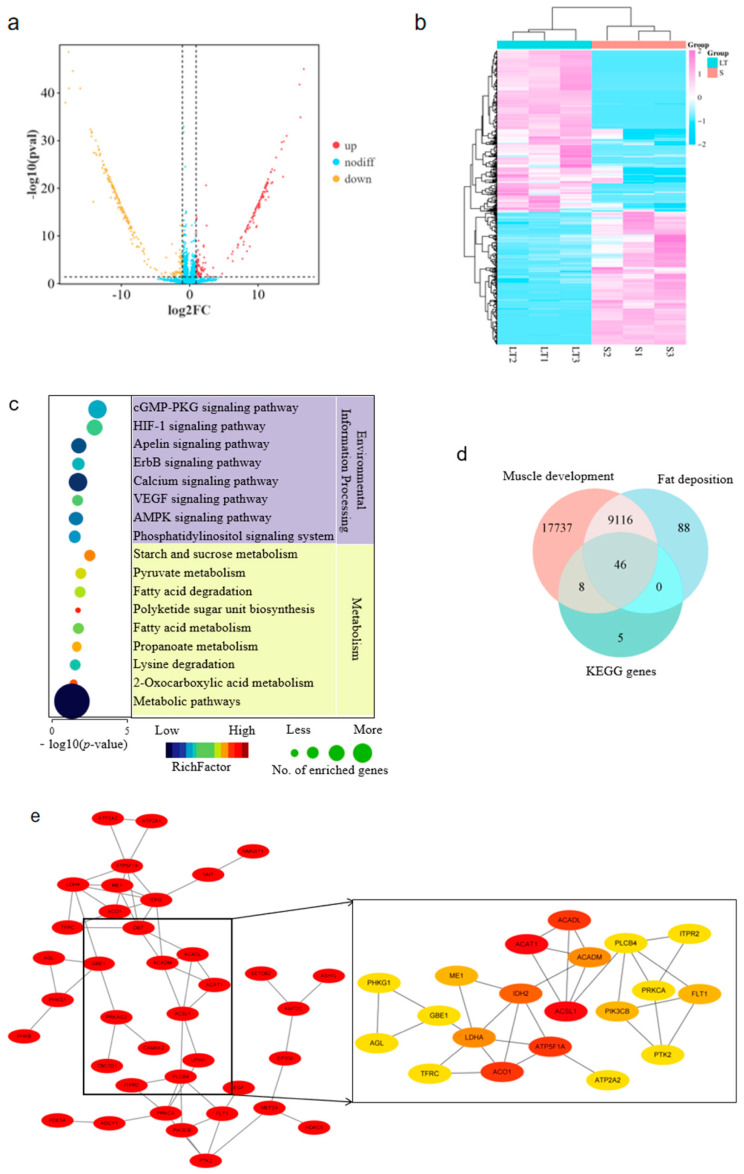
Characterization of differentially expressed (DE) circRNAs. (**a**) Volcano plot of DE circR-NAs; (**b**) Heat map of DE circRNAs; (**c**) KEGG enrichment of DE circRNAs; (**d**) Venn diagram of genes enriched in (**c**), and those associated with fat deposition and muscle development as defined by Genecard; (**e**) Protein–protein interaction of intersectional genes identified in (**d**).

A total of 441 DE circRNAs were identified, including 199 up- and 242 down-regulated in LT compared to ST muscles (Figure 1a,b; Table S2). The DE circRNAs are generated from 347 genes. These parent genes were subject to KEGG enrichment and pathways included in environmental information processing and metabolism are given in Figure 1c. It was found that various pathways related to meat quality were enriched, including the cGMP-PKG signaling pathway, HIF-1 signaling pathway, propanoate metabolism, and fatty acid metabolism. A total of 59 genes were involved in the above environmental information processing and metabolism categories. Then, genes associated with fat deposition and muscle development were downloaded from Genecard (https://www.genecards.org; accessed on 2 December 2024). A total of 46 intersectional genes were identified among the 59 KEGG genes, and the fat- and muscle-associated genes (Figure 1d). PPI analysis showed that long-chain acyl CoA synthetase 1 (ACSL1) is a core gene of the 46 genes (Figure 1e). Circ-06958 is produced by the ACSL1 gene and is highly expressed in both muscles. Therefore, Circ-06958 was selected for further analysis.

### 3.2. Circ-06958 Identification and Subcellular Localization

**Figure 2:**
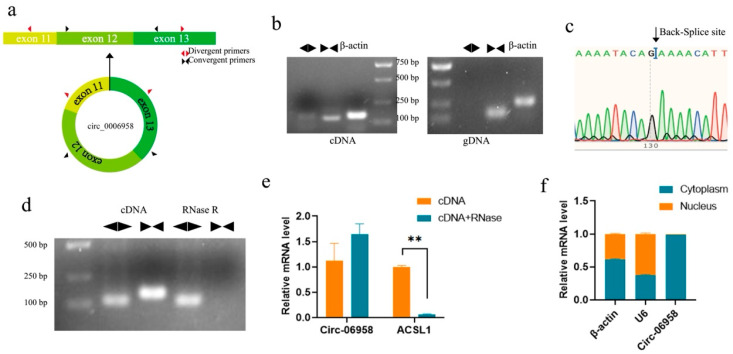
Characterization of Circ-06958 in porcine skeletal muscles. (**a**) Schematic structure of Cric-06958; (**b**) PCR products amplified with divergent and convergent primers in cDNA and gDNA; (**c**) Validation of back splice junction by Sanger sequencing; (**d**,**e**) Confirmation of closed-loop characteristics with RNase R digestion with agarose gel electrophoresis (**d**) and qPCR (**e**), respectively; (**f**) Quantification of Cric-06958 in the nucleus and cytoplasm. **, *p <* 0.01; the same as below.

According to the results of RNA-seq, Circ-06958 was derived from the exon 11–13 of the porcine *ACSL1* gene, with a total length of 348 bp (Figure 2a). Convergent and divergent primers were first designed to validate the sequence of Circ-06958 with PCR. As shown in Figure 2b, both the primers could obtain a single target band when cDNA was used as a template for amplification, while no band was obtained by divergent primers when gDNA was used as a template, indicating that there is a circular sequence in the cDNA of Circ-06958. Sequencing analysis confirmed the presence of a splice junction in the products obtained with the divergent primers (Figure 2c).

Next, RNase R digestion resulted in the disappearance of products amplified with convergent primers, while it had no effect on the amplification of divergent primers (Figure 2d). qPCR further confirmed that the expression level of Circ-06958 was not changed after RNase R treatment, while the level of linear mRNA was significantly decreased (Figure 2e). Then, it was shown that Circ-06958 was mainly expressed in the cytoplasm, as revealed by quantitative analysis in nucleus and cytoplasm separately, suggesting it plays a regulatory role as ceRNA (Figure 2f).

### 3.3. CircRNA-06958 Enhances Cell Proliferation by Promoting Cell Cycle Progression

**Figure 3:**
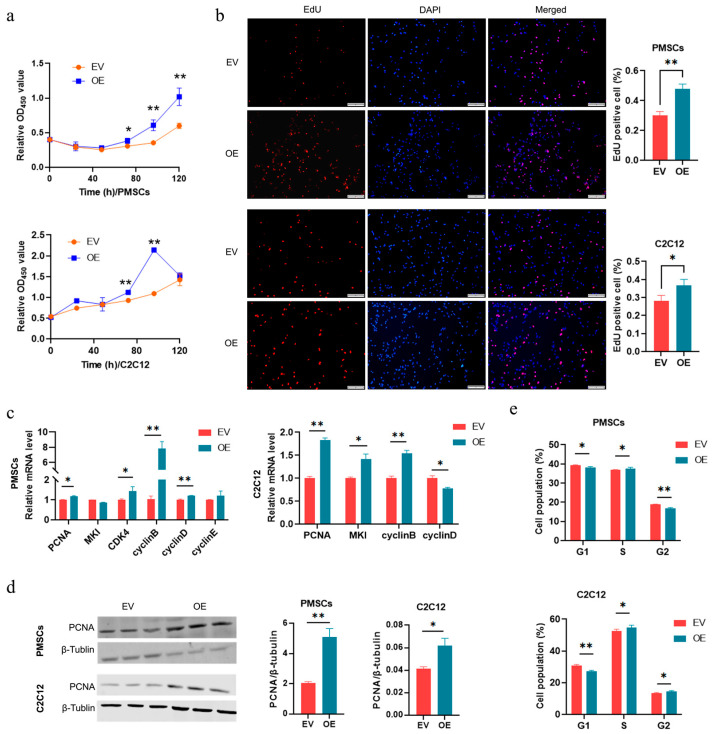
Circ-06958 increases cell proliferation by promoting cell cycle progression. (**a**) CCK-8 assays; (**b**) EdU assays. The bar is 200 μm; (**c**) Quantification of marker genes of cell proliferation; (**d**) Western blotting for PCNA; (**e**) Cell cycle distribution analyses. EV and OE indicate that the cells were treated with an empty vector, pCD2.1-CIR, and plasmids overexpressing Circ-06958, respectively. *, *p <* 0.05; **, *p <* 0.01; the same as below.

To reveal the role of Circ-06958, plasmids overexpressing Circ-06958 were constructed successfully (Figure S1a). We first analyzed the effects of Circ-06958 on the proliferation of PMSCs. CCK-8 assay showed that overexpression of Circ-06958 (OE) increased the cell viability significantly compared with the control group (EV) (*p <* 0.01; Figure 3a). EdU assay further showed that ectopic Circ-06958 increased the cell number significantly (*p <* 0.01; Figure 3b). Ectopic Circ-06958 increased the mRNA level of Proliferating Cell Nuclear Antigen (*PCNA)* (*p <* 0.05), Cyclin-dependent kinase 4 (*CDK4)* (*p <* 0.05), Cell cycle protein B (*cyclin B*) (*p <* 0.01), and Cell cycle protein D (*cyclin D*) (*p <* 0.01) significantly (Figure 3c). At the same time, the protein level of PCNA was significantly increased by Circ-06958 overexpression (*p <* 0.01; Figure 3d). Flow cytometry analyses showed that Circ-06958 decreased cell number in G0/G1 (*p <* 0.01) and increased it in the G2/M phase (*p <* 0.01) significantly (Figure 3e and Figure S2a).

Mouse myoblast, C2C12 cells, was then used to validate the promoting effects of Circ-06958 on cell proliferation. As observed in PMSCs, ectopic Circ-06958 could increase the cell viability (*p <* 0.01; Figure 3a), cell number (*p <* 0.05; Figure 3b), mRNA level of cell proliferation marker genes including *PCNA* (*p <* 0.01), proliferation marker protein Ki-67(*MKI*) (*p <* 0.05), *cyclin D* (*p <* 0.01) and *cyclin B* (*p <* 0.05; Figure 3c), and the protein level of PCNA (*p <* 0.05; Figure 3d). Also, the cell cycle progression was promoted by overexpressing Circ-06958 in C2C12 cells (Figure 3e and Figure S2a). These results indicate Circ-06958 could enhance the proliferation of muscle cells by promoting cell cycle progression.

### 3.4. Construction of Regulatory Axis Among CircRNA-06958, miR-31-5p, and AK4

**Figure 4:**
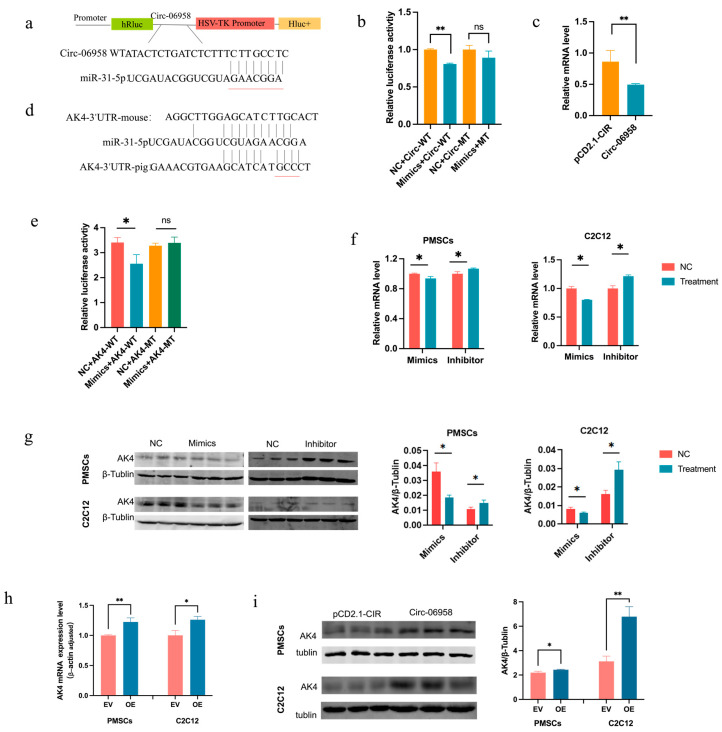
Construction of Circ-06958/miR-31-5p/AK4 axis. (**a**) Schematic structure of reporter genes containing Circ-06958. The underlined sequences were deleted in mutant type (MT) reporters. WT: wild type; (**b**) Effects of miR-31-5p on the expression of reporter genes containing Circ-06958; (**c**) Effects of Circ-06958 overexpression on the endogenous expression of miR-31-5p; (**d**) Putative binding site of miR-31-5p in 3’ UTR of *AK4* gene. The underlined sequences were deleted in MT reporters containing *AK4* 3’ UTR; (**e**) Effects of miR-31-5p on the expression of reporter genes containing *AK4* 3’ UTR; (**f**,**g**) Effects of miR-31-5p on the endogenous expression of *AK4* at mRNA (**f**) and protein (**g**) level. NC: negative control; effects of Circ-06958 on the endogenous expression of *AK4* at mRNA (**h**) and protein (**i**) level. EV and OE indicate that the cells were treated with empty vector, pCD2.1-CIR, and plasmids overexpressing Circ-06958. The same as below. ns, no significant difference; *, *p <* 0.05; **, *p <* 0.01; the same as below.

Subcellular localization analysis showed that Circ-06958 might function as ceRNA. To reveal the mechanisms through which Circ-06958 regulates cell proliferation, the miRNA targeted by Circ-06958 was identified. A binding site for miR-31-5p was predicted with the online software circAtlas 3.0 (https://ngdc.cncb.ac.cn/circatlas/ (accessed on 9 January 2024)) and Circular-RNA-Interactome (https://circinteractome.nia.nih.gov/index.html (accessed on 11 Jan 2024)) (Figure 4a). Dual-luciferase reporter gene analyses showed that miR-31-5p mimics could decrease the luciferase activities of reporter genes containing Circ-06958 sequence, at the same time deletion of the putative binding site abolished the suppressing effects of mimics on luciferase activities (Figure 4b). Moreover, overexpression of Circ-06958 reduced the expression of endogenous miR-31-5p in PMSCs (Figure 4c). These results indicated that Circ-06958 could absorb miR-31-5p directly.

Then, 284 potential target genes of miR-31-5p were predicted by the online software TargetScan (https://www.targetscan.org/vert_80/ (accessed on 28 January 2024)) and miRbase (https://www.mirbase.org (accessed on 28 January 2024)). Among them, *AK4*, targeted by miR-31-5p in both pigs and mice (Figure 4d), was selected for further analysis. As shown in Figure 4e, miR-31-5p overexpression weakened the expression of luciferase genes, and deletion of the putative binding site abolished the suppressing effects of miR-31-5p mimics on the expression of reporter genes. Furthermore, miR-31-5p inhibited the endogenous expression of *AK4* gene at both mRNA and protein levels in all the two cells studied, PMSCs and C2C12 (Figure 4f,g); however, overexpression of Circ-06898 promoted the endogenous expression of *AK4* genes at mRNA and protein levels (Figure 4h,i). These results together indicate that Circ-06898 can inhibit the degradation of *AK4* by miR-31-5p.

### 3.5. miR-31-5p Inhibits Cell Proliferation by Suppressing Cell Cycle Progression

**Figure 5:**
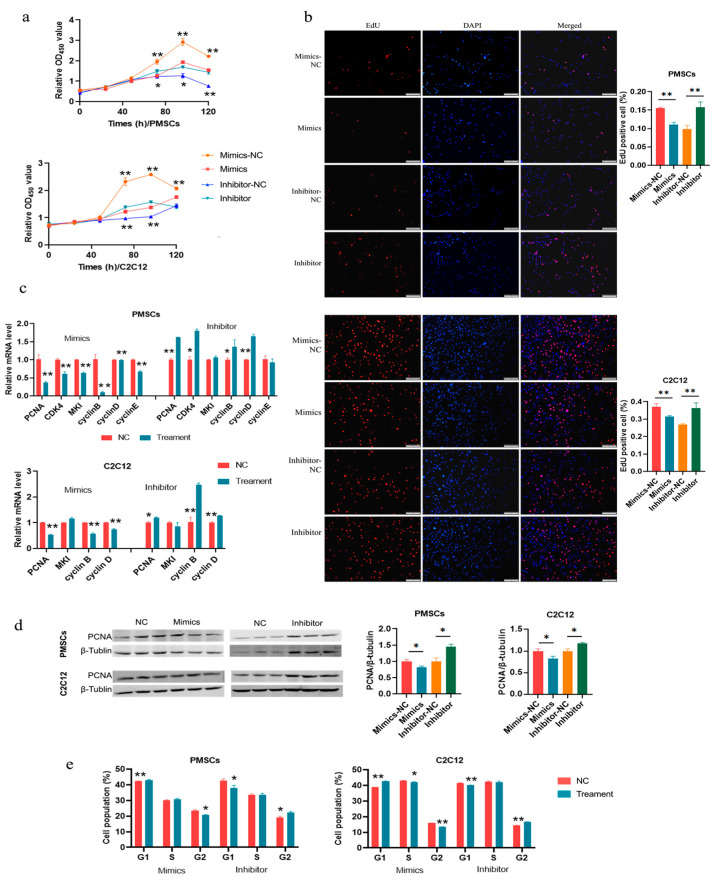
miR-31-5p inhibits cell proliferation via G0/G1 cell cycle arrest. (**a**) CCK-8 assays; (**b**) EdU assays. The bar is 200 μm; (**c**) qPCR analyses for cell proliferation marker genes; (**d**) Western blotting for PCNA; (**e**) Cell cycle distribution analyses. *, *p <* 0.05; **, *p <* 0.01; the same as below.

Next, we analyzed the effects of miR-31-5p on cell proliferation. MiR-31-5p mimics reduced the cell viability and the cell number, while the inhibitor increased them, as revealed by CCK-8 and EdU assays, respectively, in all the two cell types (Figure 5a,b). The mRNA expression of *PCNA*, *CDK4*, *cyclin B*, and *cyclin D* was inhibited by miR-31-5p mimics, while increased by the inhibitor (Figure 5c). The level of PCNA protein showed the same change as that of mRNA in response to miR-31-5p mimics and inhibitor (Figure 5d). Cell cycle analyses showed that the mimics increased cell number in the G0/G1 phase, and decreased that in the G2/M phase, while opposite results were obtained in cells treated with the inhibitor (Figure 5e and Figure S2b). The results showed that overexpression of miR-31-5p inhibited cell entry into the cell cycle.

### 3.6. Circ-06958 Promotes Cell Proliferation by Absorbing miR-31-5p

**Figure 6:**
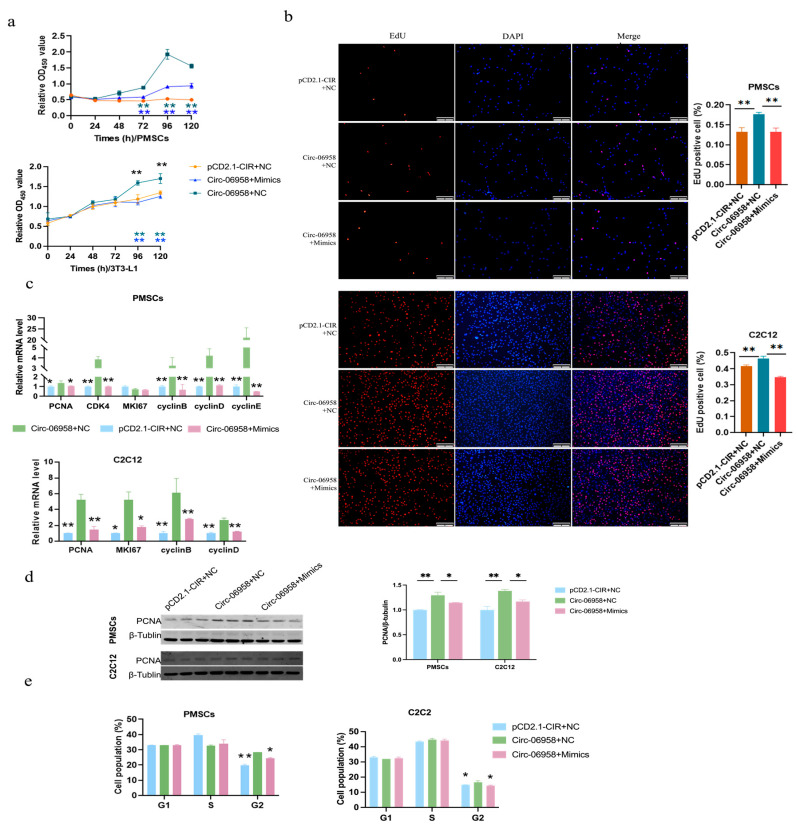
Circ-06958 promotes cell proliferation by absorbing miR-31-5. (**a**) CCK-8 assays. Dark blue asterisks indicate the differences between groups pCD2.1-CIR+NC and Circ-06958+NC, while bright blue ones indicate the differences between groups Circ-06958+NC and Circ-06958+Mimics; (**b**) EdU assays. The bar is 200 μm; (**c**) qPCR analyses for cell proliferation marker genes; (**d**) Western blotting for PCNA; (**e**) Cell cycle distribution analyses. *, *p <* 0.05; **, *p <* 0.01; the same as below.

To investigate the relationship between Circ-06958 and miR-31-5p in regulating cell proliferation, we co-transfected Circ-06958 plasmids with miR-31-5p mimics into cells. The CCK-8 and EdU assays showed that miR-31-5p alleviated the promoting effects of miR-31-5p on cell proliferation (Figure 6a,b). The RT-qPCR showed that the promoting effects of Circ-06958 on marker genes of cell proliferation were counteracted by miR-31-5p (Figure 6c). Similar results were also found in the protein level of PCNA (Figure 6d). Additionally, miR-31-5p attenuates the promoting effects of Circ-06958 on cell cycles using flow cytometry analyses (Figure 6e and Figure S3). In summary, the data indicated that Circ-06958 could promote cell proliferation by absorbing miR-31-5p.

### 3.7. AK4 Enhances Cell Proliferation by Promoting Cell Cycle Progression

**Figure 7:**
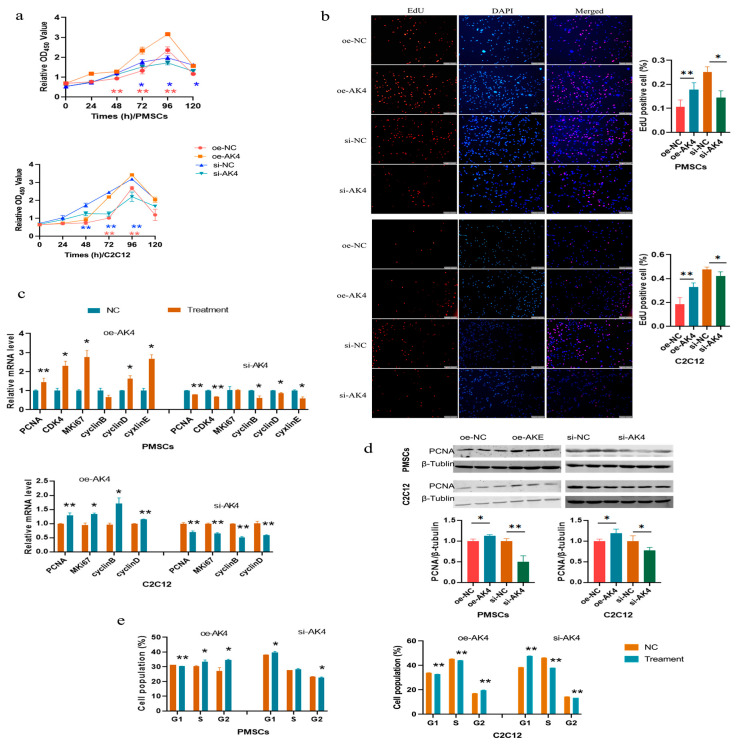
AK4 increases cell proliferation by promoting cell cycle progression. (**a**) CCK-8 assays. Red asterisks indicate the differences between oe-AK4 and oe-NC, while the blue ones indicate the differences between si-AK4 and si-NC; (**b**) EdU assays, The bar is 200 μm; (**c**) qPCR analyses for cell proliferation marker genes; (**d**) Western blotting for PCNA; (**e**) Cell cycle distribution analyses. *, *p <* 0.05; **, *p <* 0.01. oe-AK4 and si-AK4 indicate overexpression and knocking down the expression of AK4, respectively. The same as below.

To study the function of AK4 in cell proliferation, we successfully obtained plasmid overexpression and siRNA against AK4 (Figure S1c,d). Overexpression of AK4 increased the cell viability, cell number, and the mRNA level of proliferation marker genes compared to the negative control group (NC), while knocking down AK4 had opposite effects, in all the two cells (Figure 7a–c). The protein level of PCNA showed similar changes (Figure 7d). Additionally, overexpressing AK4 decreased cell population in the G0/G1 phase and increased it in the G2/M phase, and vice versa (Figure 7e and Figure S4).

### 3.8. miR-31-5p Inhibits Cell Proliferation by Silencing AK4

**Figure 8:**
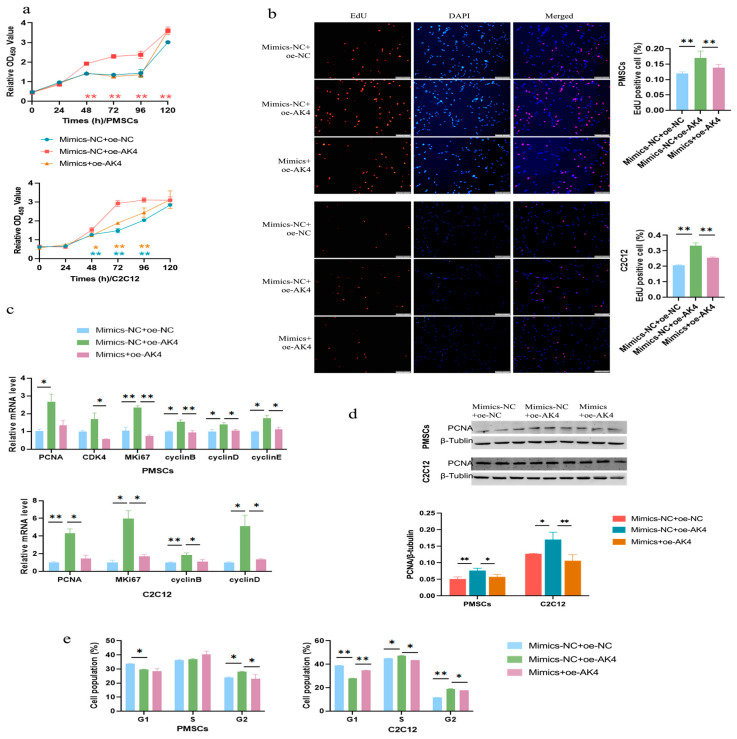
miR-31-5p inhibits cell proliferation by silencing the AK4. (**a**) CCK-8 assays. Blue asterisks indicate the differences between groups Mimics-NC+oe-NC and Mimics-NC+oe-AK4, while the yellow ones indicate the differences between groups Mimics-NC+oe-AK4 and Mimics+oe-AK4; (**b**) EdU assays. The bar is 200 μm; (**c**) qPCR analyses for cell proliferation marker genes; (**d**) Western blotting for PCNA; (**e**) Cell cycle distribution analyses. *, *p <* 0.05; **, *p <* 0.01; the same as below.

To evaluate whether miR-31-5p was involved in the regulation of cell proliferation by AK4, rescue experiments were performed. We co-transfected cells with miR-31-5p mimics and plasmids overexpressing AK4. The results of CCK-8 and EdU demonstrated that miR-31-5p inhibited the promoting effects of AK4 on cell proliferation in all two cells (Figure 8a,b). Following miR-31-5p co-transfection, AK4-enhanced expression of cell proliferation marker genes was reversed at both the mRNA and protein level (Figure 8c,d). Furthermore, consistent results were obtained in cell cycle analyses as the promoting effects of AK4 on cell cycle progression were abolished by miR-31-5p co-transfection (Figure 8e and Figure S5). In conclusion, the data indicate that miR-31-5p inhibits cell proliferation by silencing AK4.

## 4. Discussion

CircRNAs are a kind of RNA that is stable and conserved with covalently closed loop characteristics. They are involved in post-transcriptional control of gene expression via acting as a ceRNA [24], and thus play an important role in cell activity, including proliferation, migration, and differentiation [25]. However, studies on circRNAs are limited, and the number of circRNAs is far less than expected, as revealed by high-throughput sequencing. Here, we characterized a novel circRNA, Circ-06958, in muscles with differential meat quality. We further demonstrated that Circ-06958 promoted cell proliferation by the miR-31-5p/*AK4* axis in muscle cells. The results will contribute to further revealing the mechanisms underlying the differential phenotype between the longissimus thoracis and semitendinosus muscles.

Through RNA-seq performed in the longissimus thoracis and semitendinosus muscles, we identified a lot of differentially expressed circRNAs. Among them, Circ-06958 was identified as one of the most important candidates for meat quality, owing to its expression level and the role of the parent gene. The Circ-06958 was then cloned, and the reverse splice site was validated with molecular biology experiments. A series of recent research studies showed that circRNAs could regulate cell proliferation in physiological and pathological processes. For example, Circ_0110940 exerts a pro-proliferative effect in gastric cancer through the miR-1178-3p/*SLC38A6* axis [26]. Circ_0037078 promotes trophoblast cell proliferation, migration, invasion, and angiogenesis [27]. Circ-SIRT1 promotes colorectal cancer cell proliferation by recruiting and binding eIF4A3 [28]. Circ-SATB2 regulates the proliferation and differentiation of vascular smooth muscle cells by upregulating STIM1 expression and absorbing miR-939 [29].

Both muscle fiber composition and muscle development are main factors determining meat quality, and both of them are associated with muscle cell proliferation. Here, we first analyzed the role of Circ-06958 in muscle cell proliferation. It was shown that Circ-06958 increased the proliferation of PMSCs by reducing the percentage of cells in the G0/G1 phase and promoting cell cycle progression in pigs. Then, mouse C2C12 cells were used to confirm the promoting effects of Circ-06958 on cell proliferation, and consistent results were obtained. The composition of muscle fibers determines the overall biochemical and functional characteristics of muscle tissue, which, in turn, affects its quality as fresh meat [30,31]. By using the two different cell lines, we made clear that Circ-06958 is functional when conserved in regulating cell proliferation among mammals.

Many studies have found that circRNAs can function as miRNA sponges to alleviate the inhibitory effects of miRNA on mRNAs [32]. circRILPL1 can act as a sponge for miR-145 to rescue the inhibitory effect of miR-145 on the PI3K/AKT signaling pathway, thereby promoting myoblast growth [33]. CircFUT10, circFGFR4, and circ-LMO7 circRNAs affect skeletal muscle differentiation by recruiting miR-133a, miR-107, and miR-378a-3p, respectively [34,35,36]. Through separate studies on nuclei and cytoplasm, we found that Circ-06958 mainly existed in cytoplasm, indicating a potential of Circ-06958 as a ceRNA. Thereafter, regulatory axis Circ-06958/miR-31-5p/AK4 was constructed experimentally based on bioinformatic prediction. Further analysis made sure that Circ-06958 exerted promoting effects on cell proliferation via the miR-31-5p/AK4 axis.

miR-31-5p has been demonstrated to have a dual role in cell proliferation. It is down-regulated in gastric cancer and exerts an inhibitory effect by binding to the 3′-UTR of *HDAC2* [37]. In HepG2 cells, miR-31-5p inhibits cell proliferation, migration, and invasion by regulating Sp1 [38]. However, miR-31-5p is expressed at increased levels in human cervical cancer cells and clinical tissues, and accelerates tumor growth and development in vivo by inhibiting the expression of *BAP1* [39]. The role of miR-31-5p in muscle cells still remains unclear. In this study, we revealed that miR-31-5p inhibited cell proliferation via G0/G1 cell cycle arrest in two different cells, PMSCs and C2C12, indicating a stable effect of miR-31-5p on cell proliferation in normal cells.

**Figure 9:**
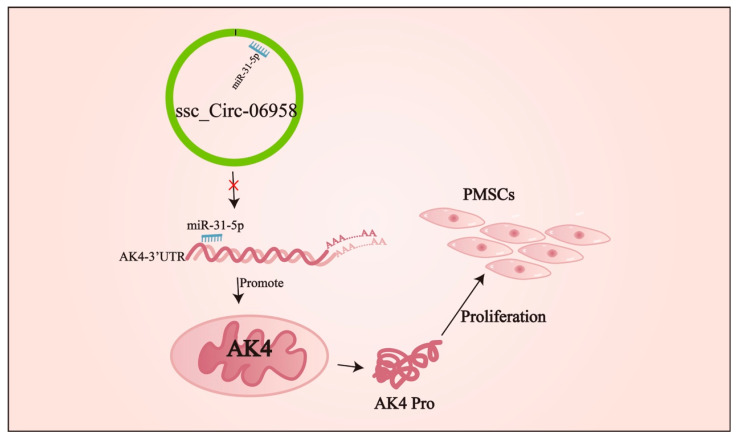
Model of Circ-06958 promoting cell proliferation by miR-31-5p/AK4 axis.

*AK4* is a unique member of the adenylate kinase family [40]. *AK4* is localized in the mitochondrial matrix and has been shown to interact with mitochondrial ADP/ATP transporters [41,42]. *AK4* can bind to mitochondrial adenine nucleotide translocator (ANT), as a stress response protein, to maintain cell physiological activity [43]. Overexpression of *AK4* can increase the level of reactive oxygen species in cells to enhance the stability of HIF-1α protein and its transcription level, which helps to control glucose metabolism and cell proliferation [44,45]; *AK4* silencing increases ATP levels in cells, thereby activating the AMPK pathway to affect fatty acid metabolism and cell proliferation [46]. Here, we showed that *AK4* promoted cell proliferation by promoting cell entry from the G0/G1 phase to the S, G2/M phase, and that miR-31-5p could attenuate the effects by directly binding to the 3’ UTR of *AK4* in multiple cells (Figure 9). The results broaden the knowledge on cell proliferation of *AK4*.

## 5. Conclusions

In summary, we characterized a differentially expressed circRNA, Circ-06958, in porcine longissimus thoracis and semitendinosus muscles based on RNA-seq results, and validated its sequence with molecular biology technology for the first time. Circ-06958 increased proliferation of cells from different tissues and species by promoting cell cycle progression, indicating it is functionally conserved in mammals. Additionally, a regulatory axis Circ-06958/miR-31-5p/AK4 was constructed experimentally. It was revealed that miR-31-5p inhibited cell proliferation, while AK4 promotes cell proliferation, by affecting cell cycle progression. Through rescue experiments, we confirmed that Circ-06958 regulated cell proliferation via the miR-31-5p/AK4 axis. This study will contribute to revealing mechanisms through which a differential phenotype was generated between the two muscles, and to controlling meat quality in pigs.

## Data Availability

Data for this article, including [Raw data of RNA-seq] are available at [China National Center for Bioinformation (cncb)] at [CRA020799; https://ngdc.cncb.ac.cn/gsa (accessed on 17 April 2023)].

## Supplementary Materials

The following supporting information can be downloaded at the following address: https://www.mdpi.com/article/10.3390/cells14181416/s1, Figure S1. Efficiencies of overexpression vector and siRNA.; Figure S2. Circ-06958 and miR-31-5p on cell cycle progression in two types of cells; Figure S3. Cell cycle detection in Circ-06958 and miR-31-5 by flow cytometry; Figure S4. Effects of AK4 on the cell cycle in cells by flow cytometry; Figure S5. Cell cycle detection in miR-31-5p and AK4 by flow cytometry. Full-length blots are presented in Figures S6–S13. Full-length gels. Table S1-A. Primers for Real-time qPCR; Table S1-B. Primers for vector construction; Table S1-C. Sequences of mimics and inhibitor of miR-31-5p, siRNAs against AK4; Table S2. Differentially expressed circRNA.

## Abbreviations

The following abbreviations are used in this manuscript:

CircRNA	Circular RNA
ceRNA	competitive endogenous RNA
RNA-seq	RNA-sequencing
ACSL1	Long-chain acyl-CoA synthetase 1
PMSCs	porcine skeletal muscle satellite cells
AK4	Adenylate Kinase 4
ncRNA	noncoding RNA
miRNAs	microRNAs
gDNA	Genomic DNA
GO	Gene Ontology
CDS	complete coding sequences
KEGG	Kyoto Encyclopedia of Genes and Genomes
CCK-8	Counting Kit 8
EdU	5-ethynyl-2′-deoxyuridine
PPI	protein–protein interaction
EV	control group
OE	overexpression of Circ-06958
NC	negative control group
PCAN	Proliferating Cell Nuclear Antigen
*CDK4*	Cyclin-dependent kinase 4
*cyclin B*	cell cycle protein B
*cyclin D*	cell cycle protein D
*MKI*	Proliferation marker protein Ki-67
