# Supplementary material for: Circ-06958 Is Involved in Meat Quality by Regulating Cell Proliferation Through miR-31-5p/AK4 Axis in Pigs
Source: Cells. 2025 Sep 10;14(18):1416. doi: 10.3390/cells14181416 (PMC12468310; doi:10.3390/cells14181416)
Supplement: Supplementary file 1 [file cells-14-01416-s001.zip › cells-3800717-supplementary.pdf]

Supplementary Materials:

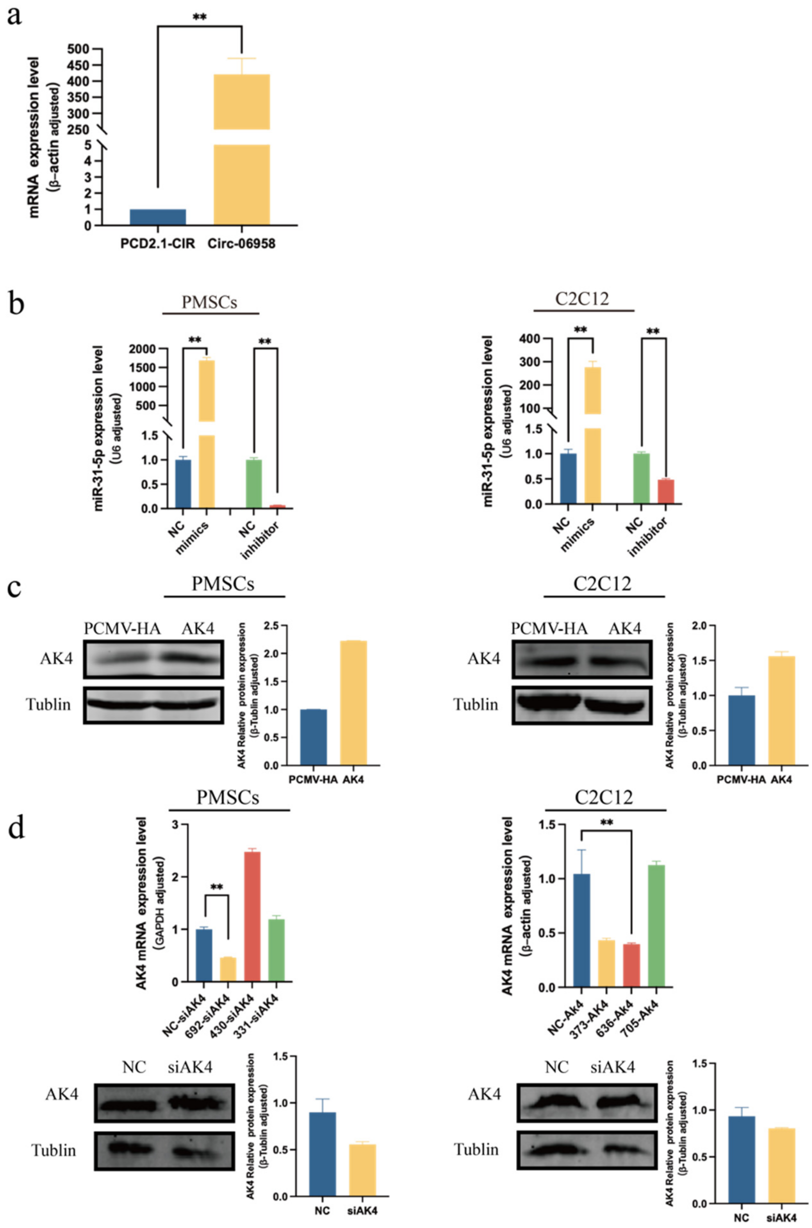

Figure S1. Efficiencies of overexpression vector and siRNA. (a) Efficiencies of vectors overexpressing Circ-06958. (b) efficiencies of mimics and inhibitor of miR-31-5p. (c) Efficiencies of vectors overexpressing AK4 by protein. (d) Efficiencies of siRNA against AK4 by mRNA and protein.

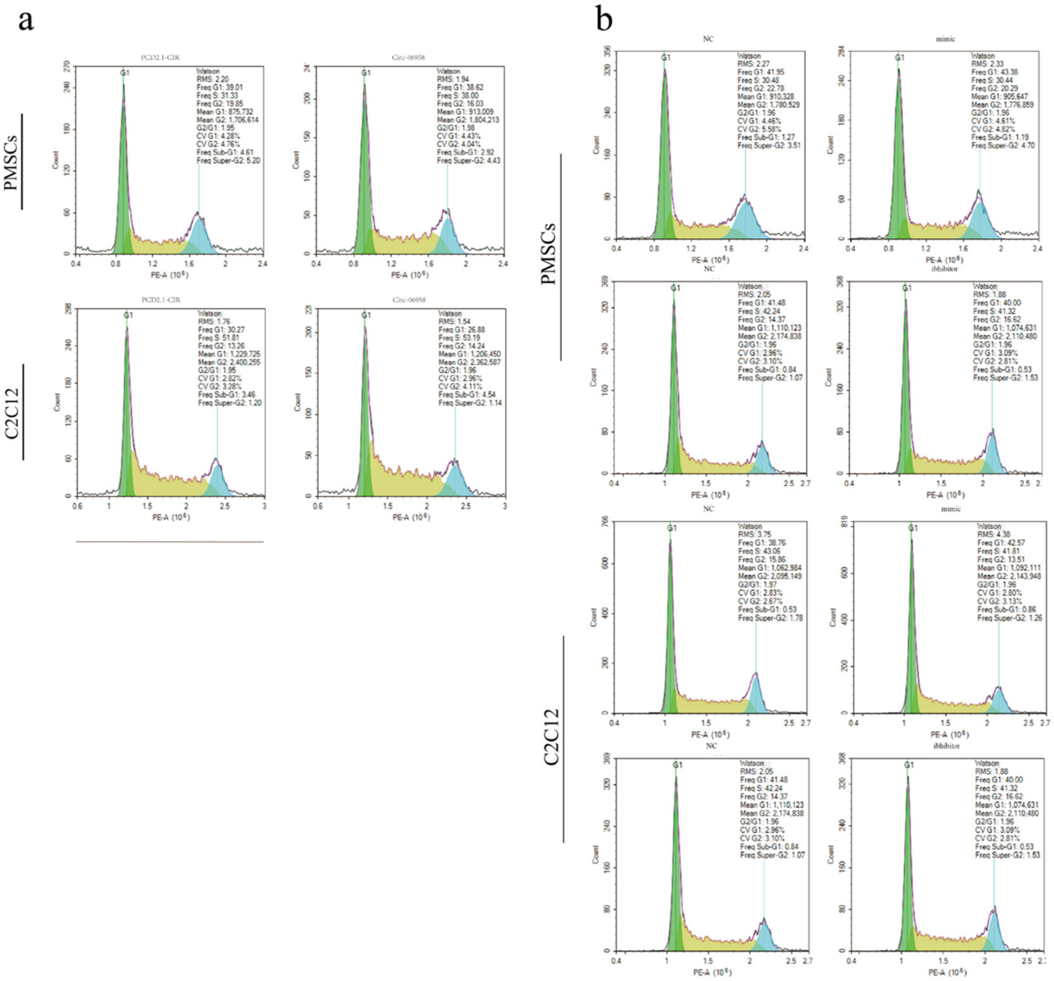

Figure S2. Circ-06958 and miR-31-5p on cell cycle progress in three types of cells. (a) Circ-06958 on cell cycle progress in three types of cells. (b) miR-31-5p on cell cycle progress in three types of cells.

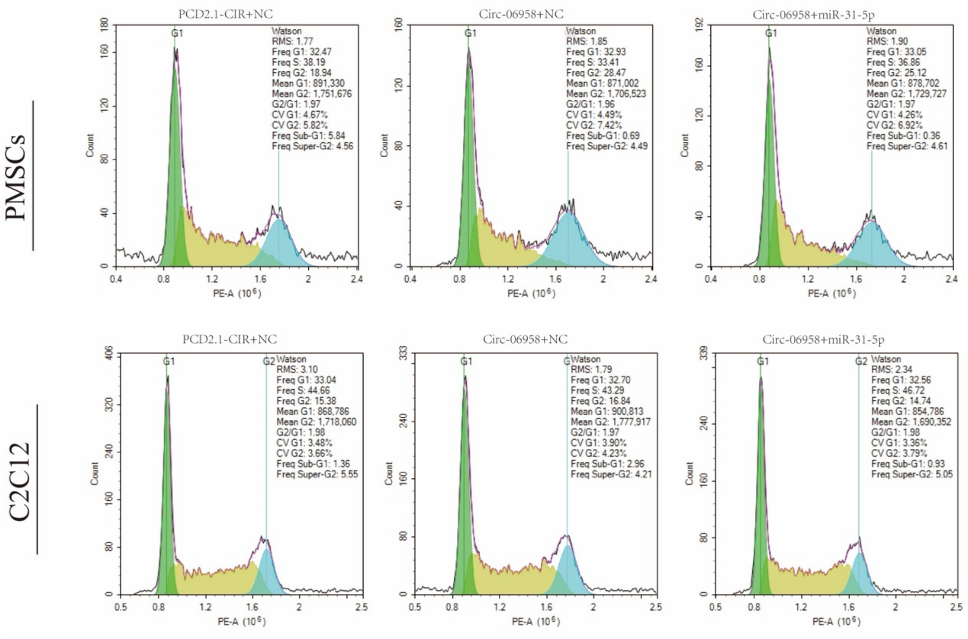

Figure S3. Cell cycle detection in Circ-06958 and miR-31-5 by flow cytometry.

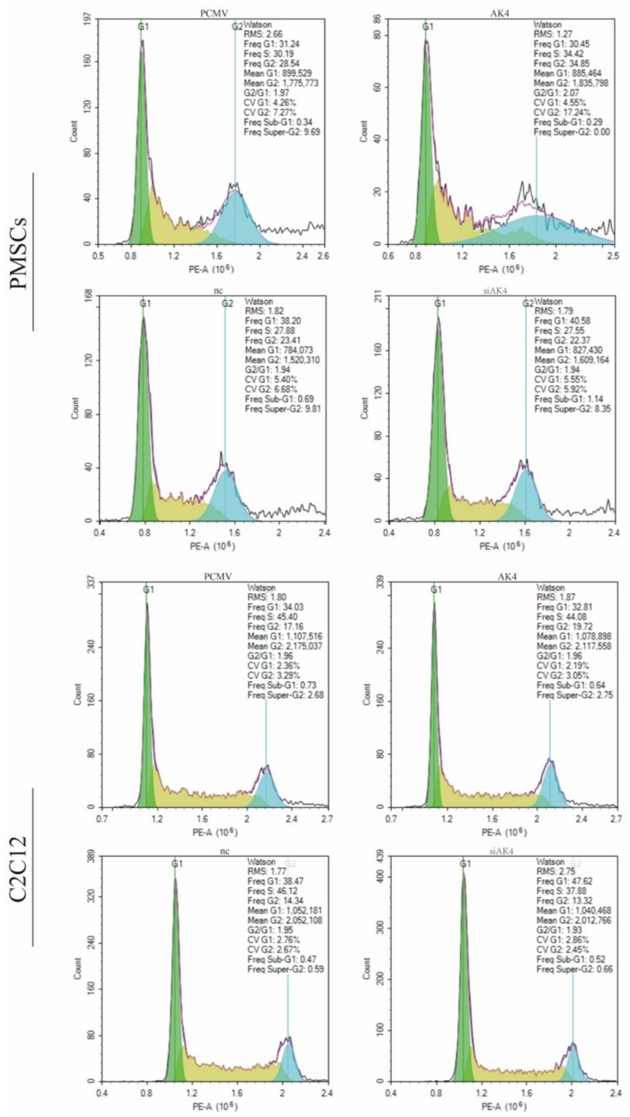

Figure S4. Effects of AK4 on cell cycle in cells by flow cytometry.

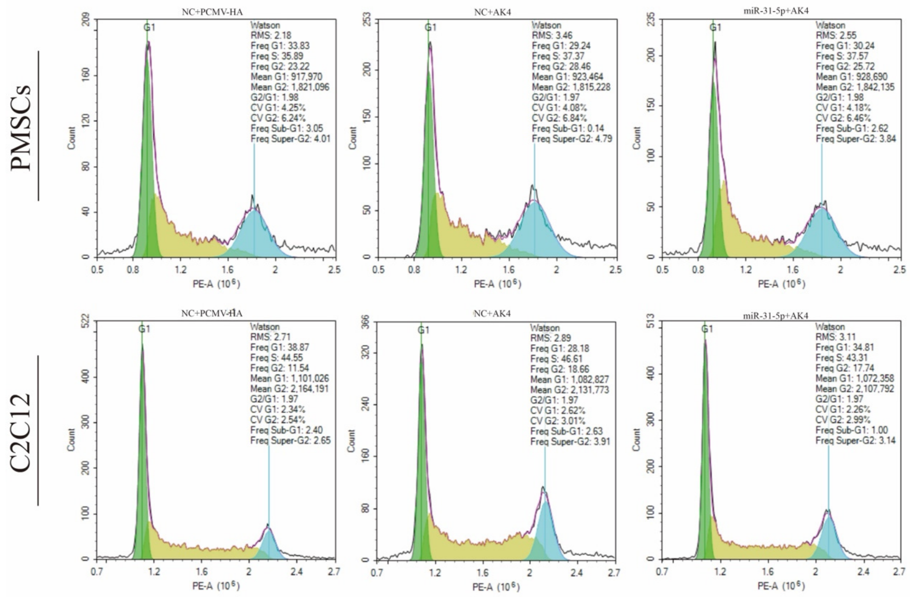

Figure S5. Cell cycle detection in miR-31-5p and AK4 by flow cytometry.

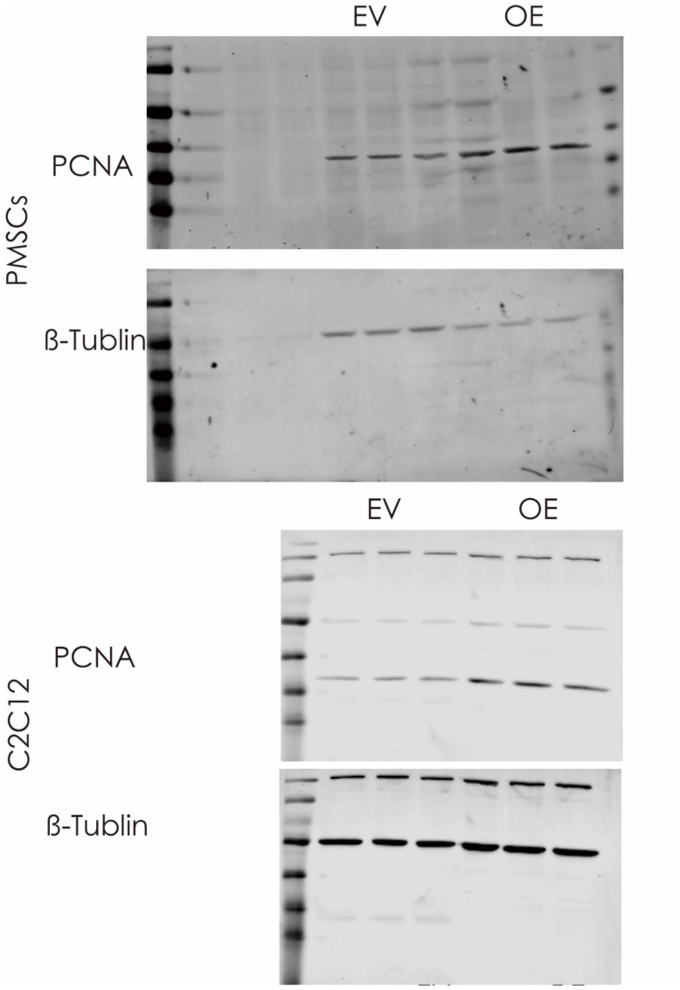

Figure S6. Circ-06958 increases cell proliferation by promoting cell cycle progression. Western blotting for PCNA.

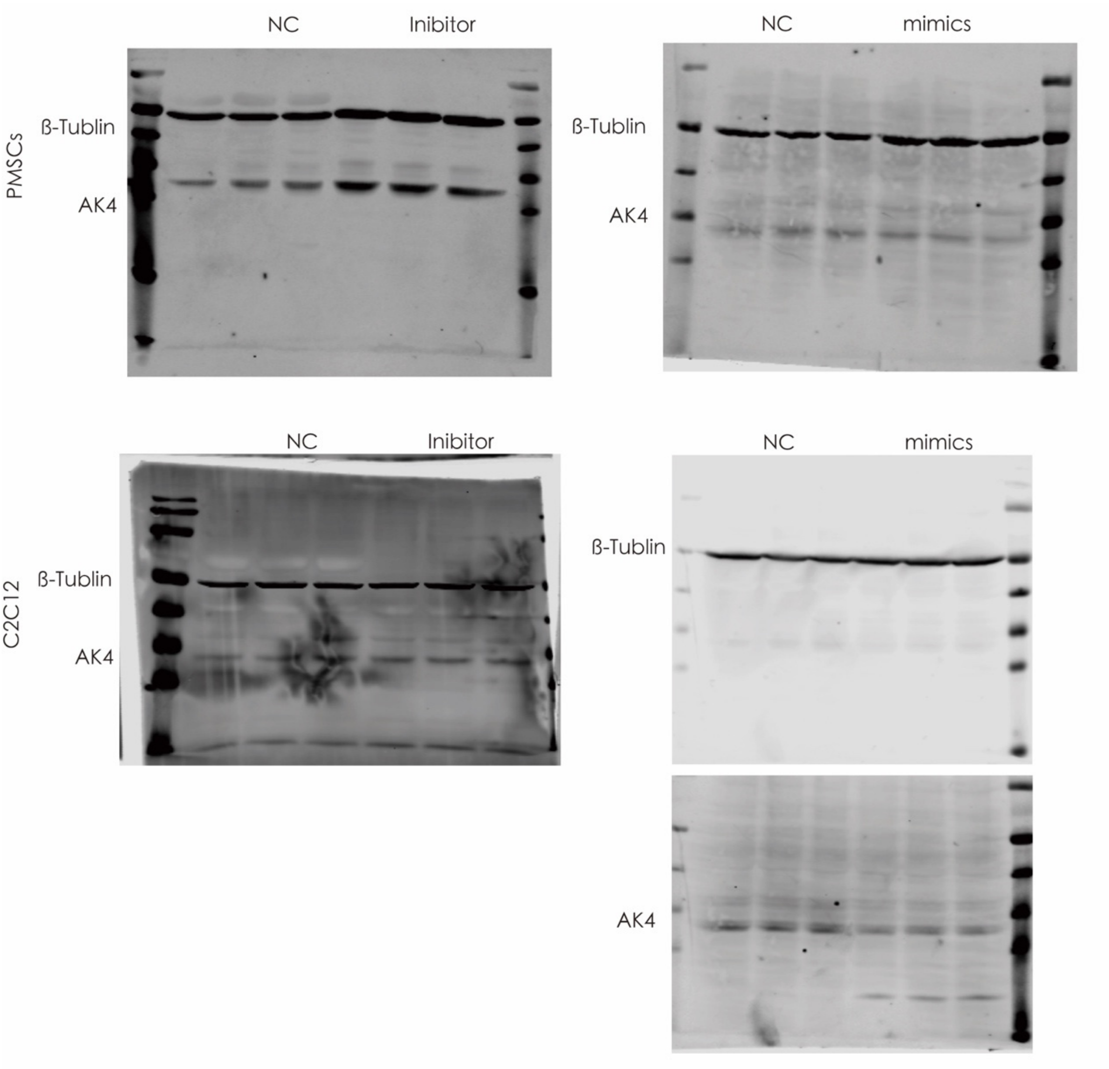

Figure S7. Effects of miR-31-5p on the endogenous expression of AK4 at mRNA.

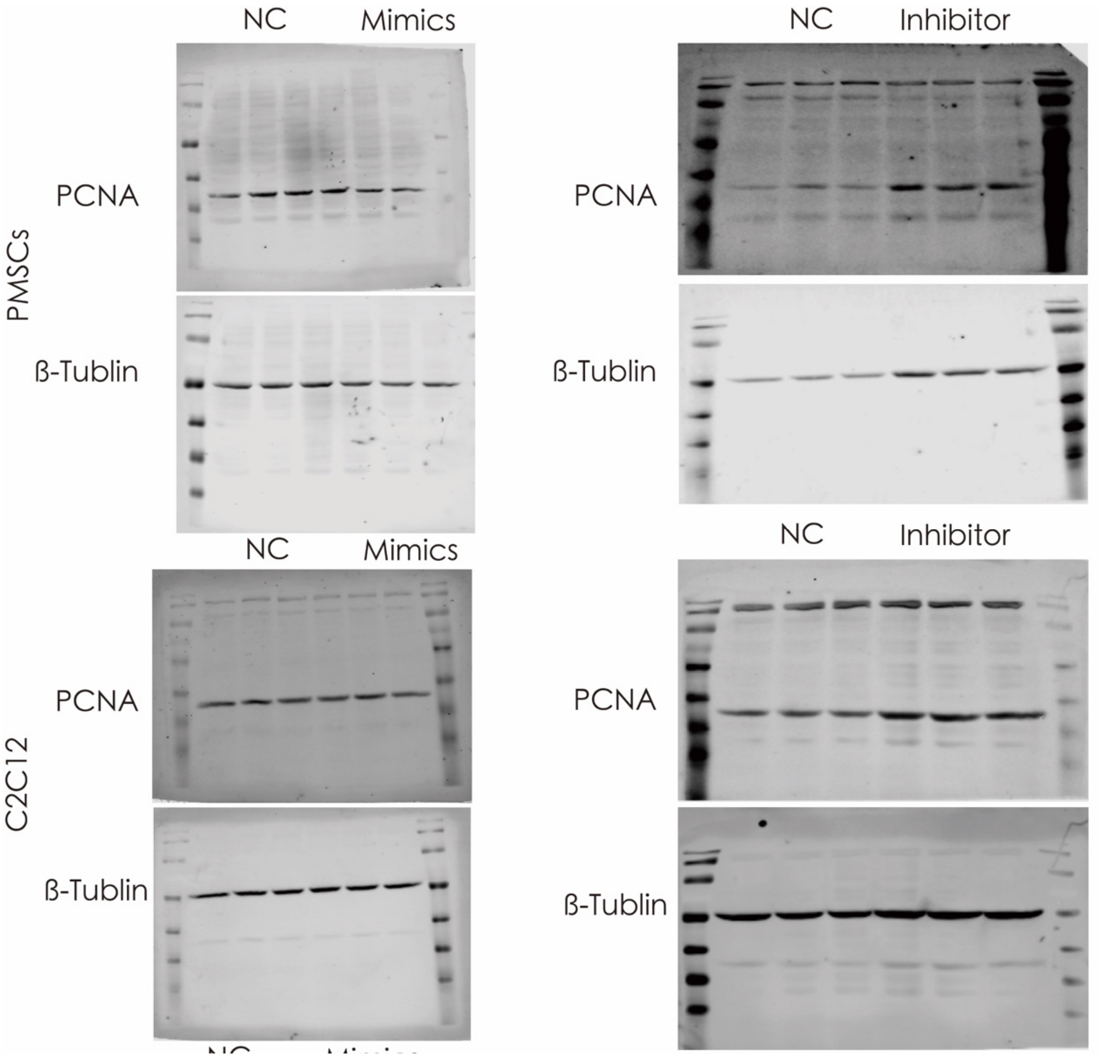

Figure S8. miR-31-5p inhibits cell proliferation via G0/G1 cell cycle arrest. Western blotting for PCNA.

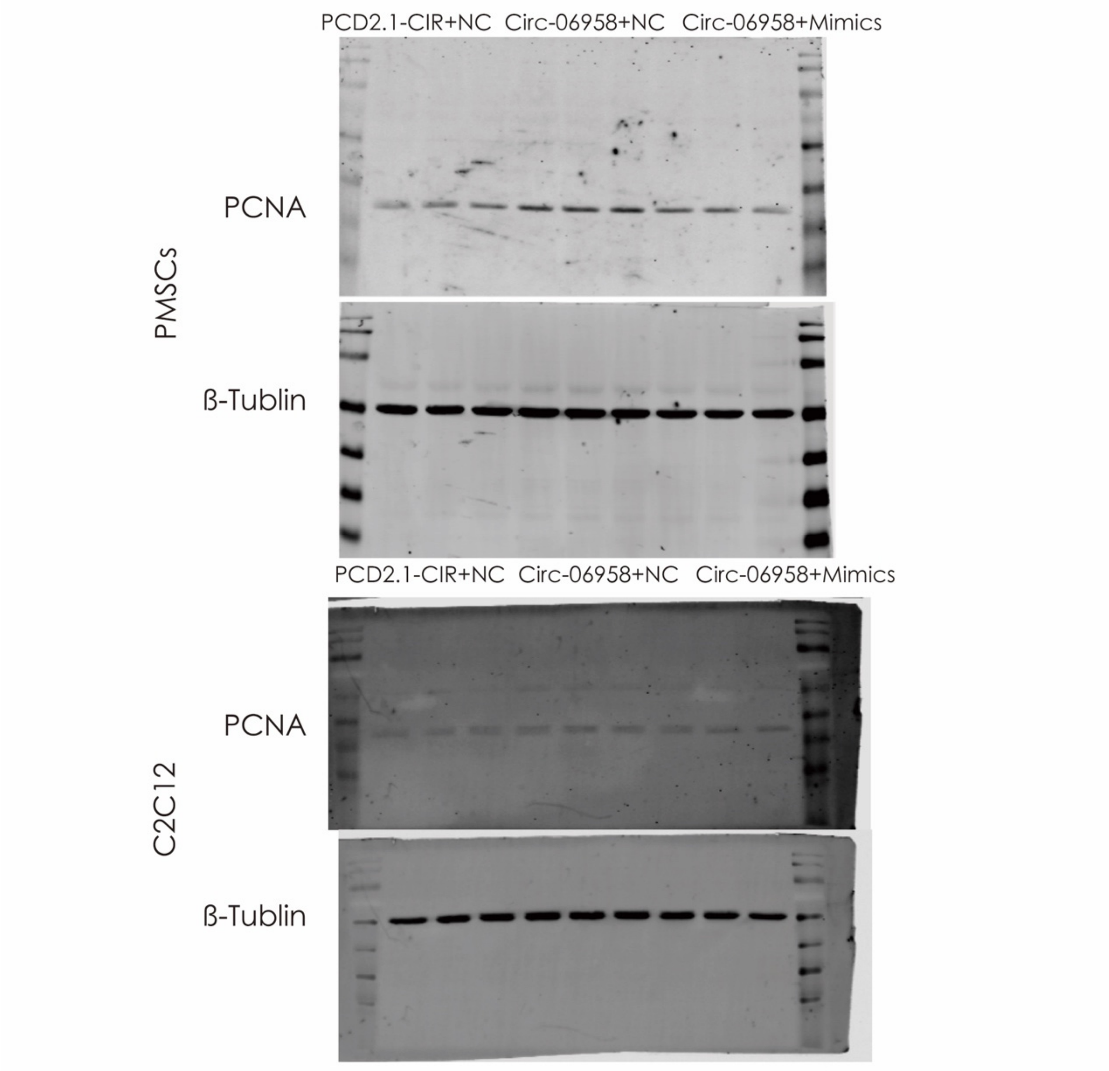

Figure S9. Circ-06958 promotes cell proliferation by absorbing miR-31-5. Western blotting for PCNA.

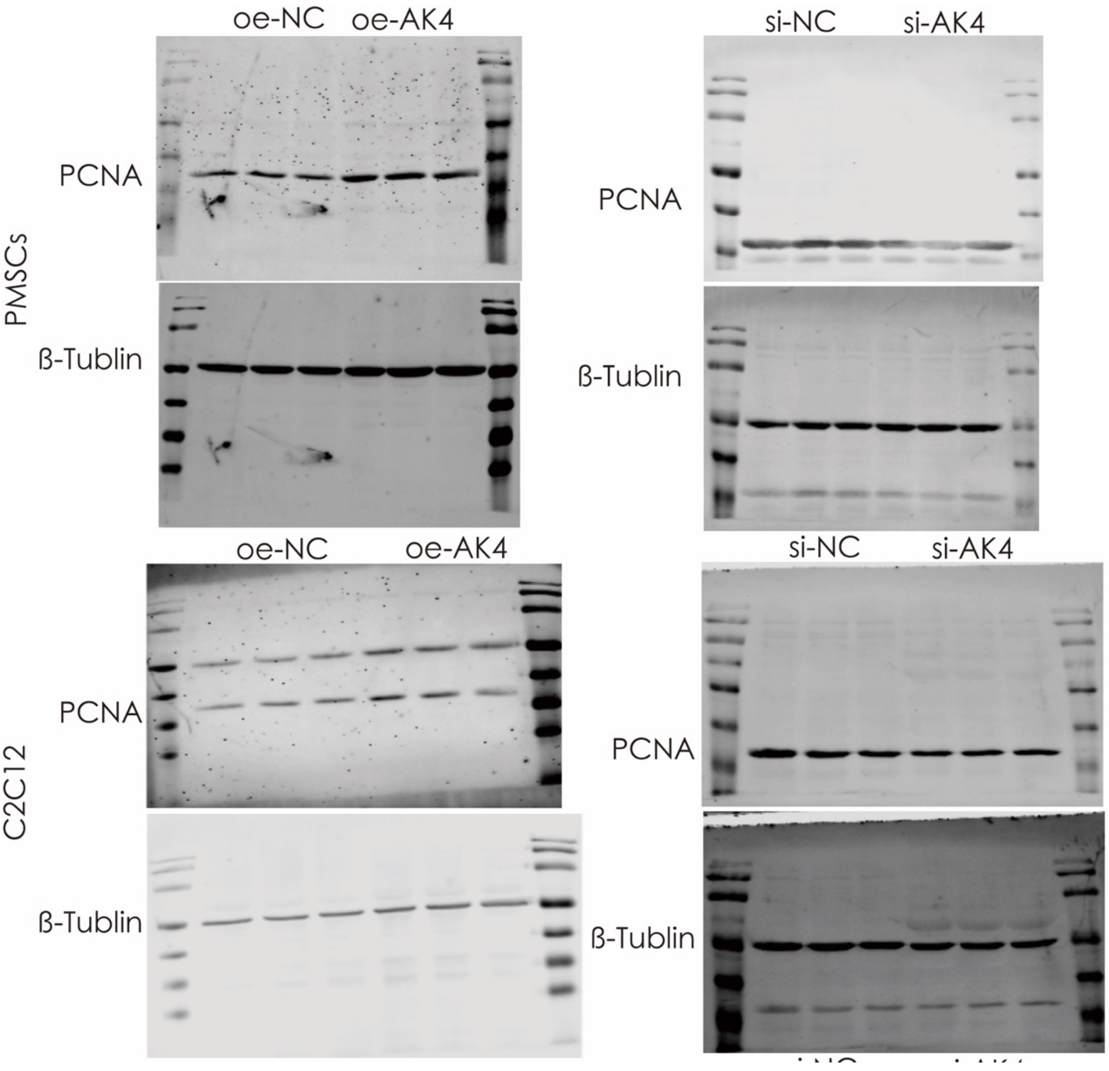

Figure S10. AK4 increases cell proliferation by promoting cell cycle progression. Western blotting for PCNA.

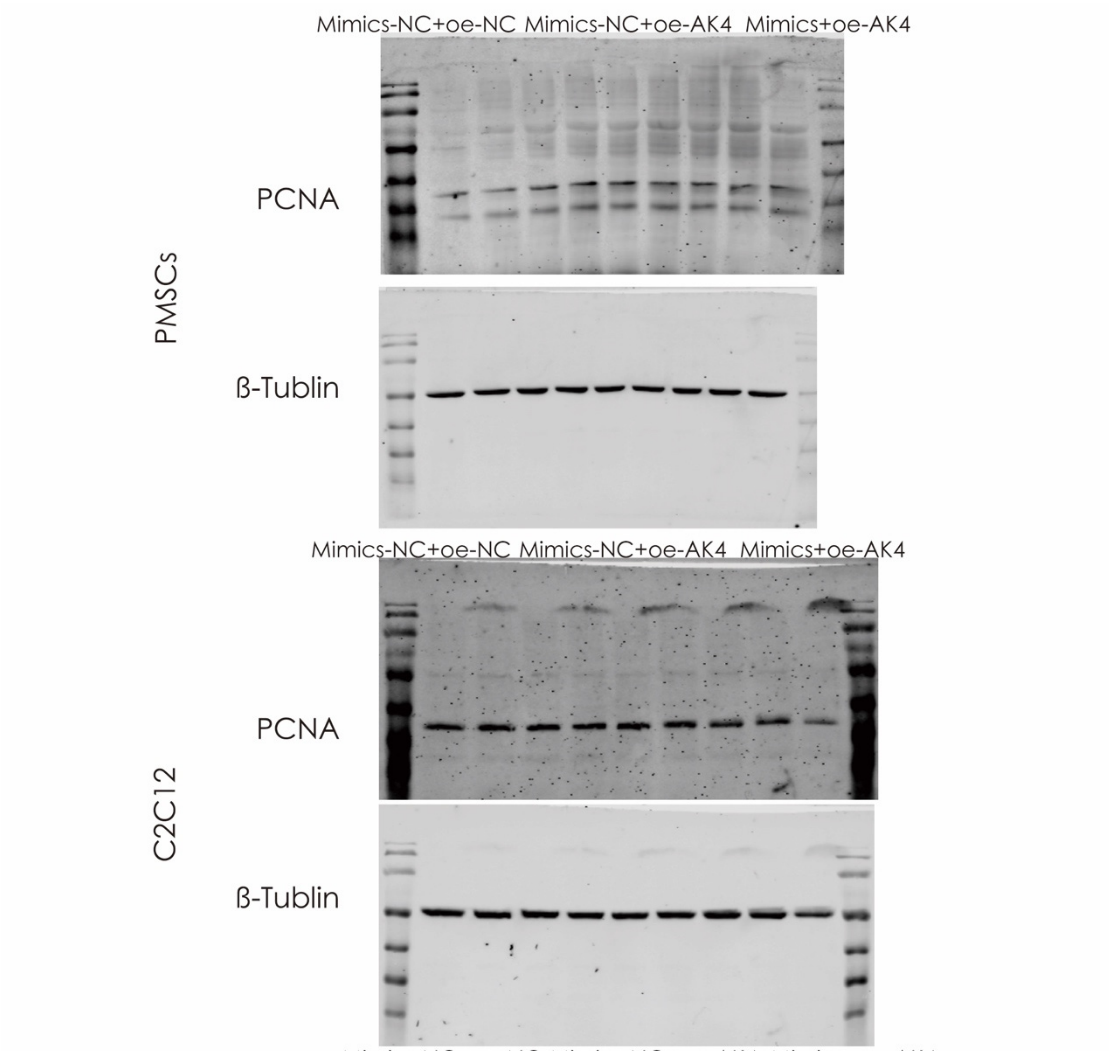

Figure S11. miR-31-5p inhibits cell proliferation by silencing the AK4. Western blotting for PCNA.

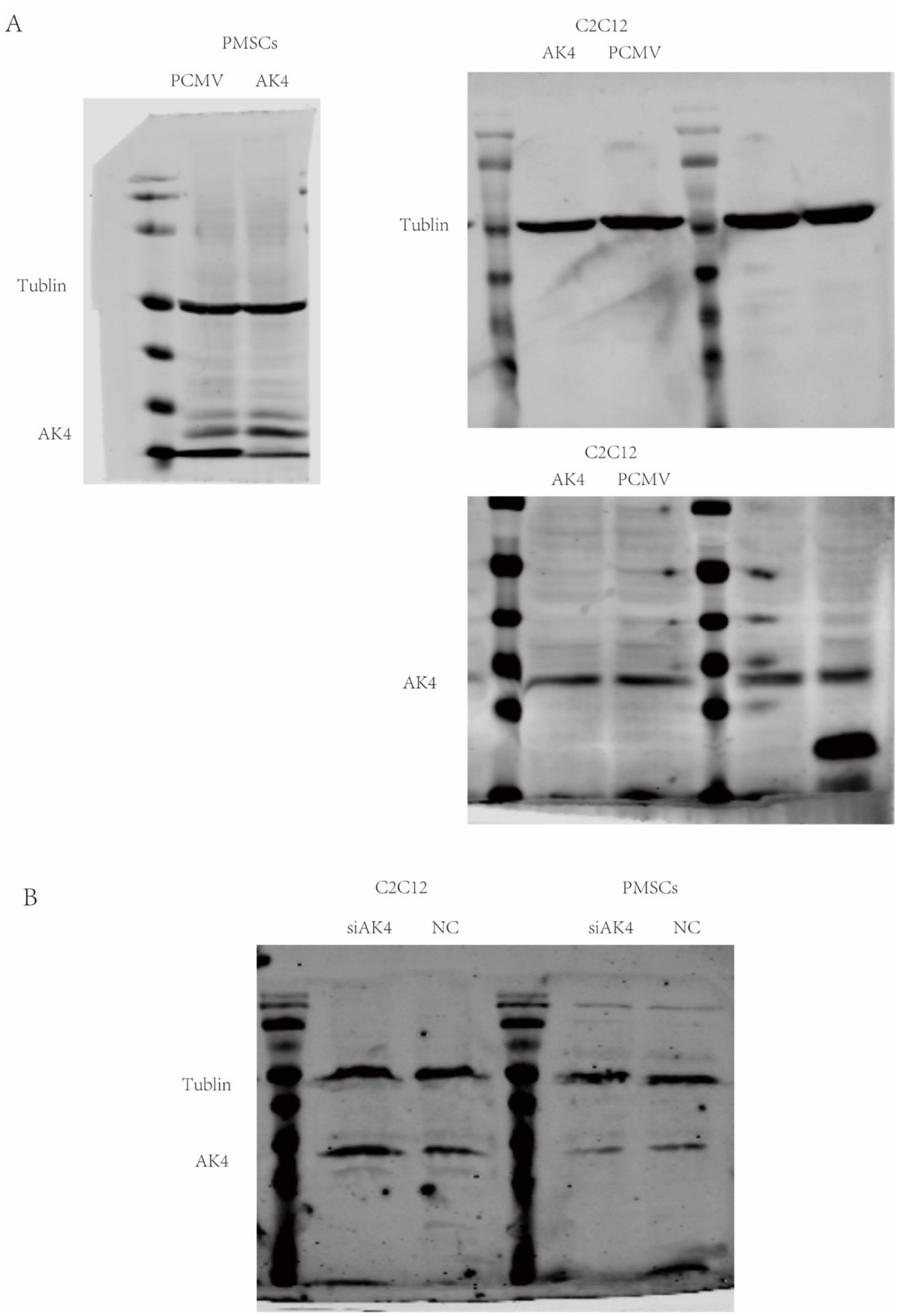

Figure S12. A. Efficiencies of vectors overexpressing AK4 by protein; B. Efficiencies of siRNA against AK4 by protein.

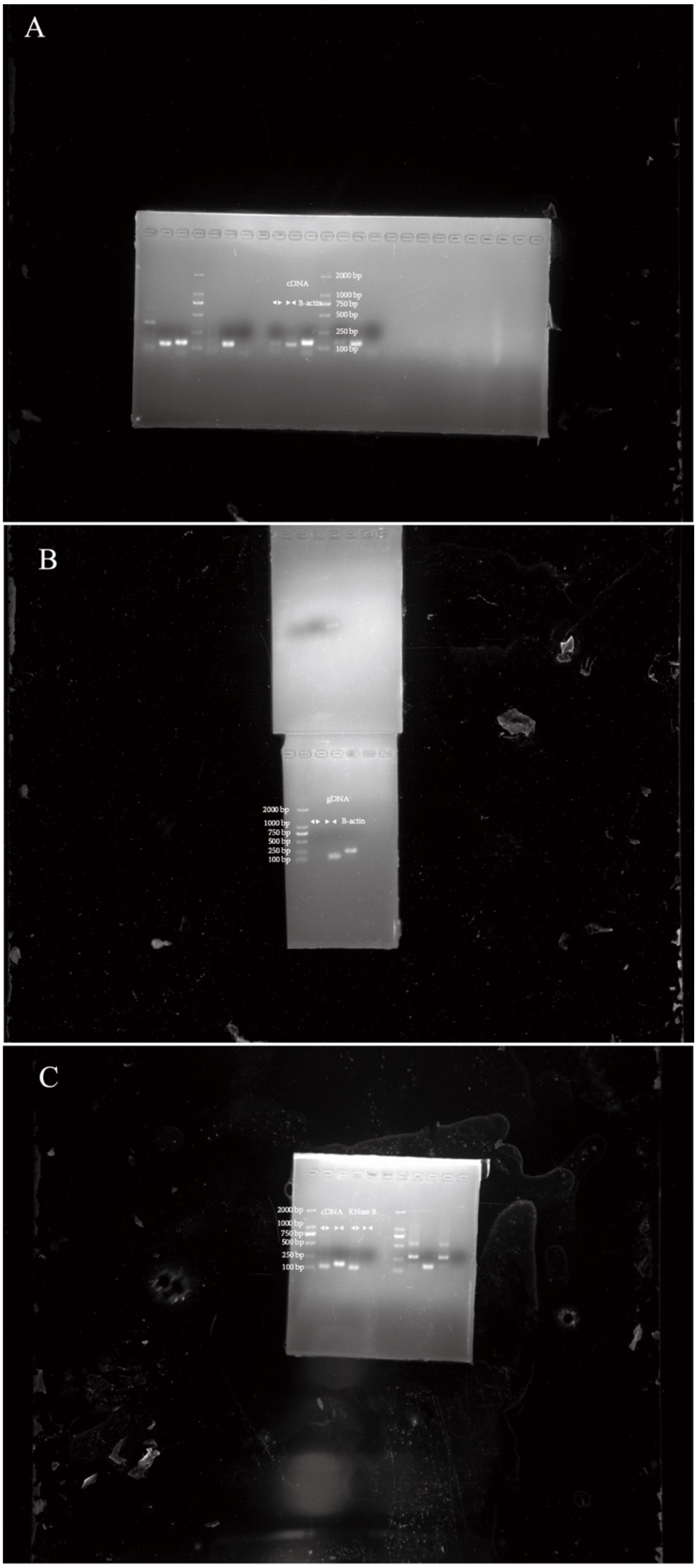

Figure S13. PCR products amplified with divergent and convergent primers in cDNA (A) and gDNA (B); (C) Confirmation of closed-loop characteristics with RNase R digestion with agarose gel electrophoresis.

Table S1-A. Primers for Realtime-qPCR

Names	Sequence (5′-3′)
Circ-06958-DiV	F: TCCAAGAGGAAGGAAGCTGAG<br>R: ATATGGGCGAGAGGCAAGA
Circ-06958-Con	F: GTTTGAGAGAGTTGTAGAGTGTGTA<br>R: GGCGAAGTCCAACAGCCA
Circ-06958-Conj	F: TGTGTAATGTTGTGTCATGGAGCTA<br>R: TCAAACATCCGGTTCAGCAGTC
Circ-06958-RT-qPCR	F: GGACAAGCAAATACCACGC<br>R: ATCGGAGGAAGGACTGAATG
PCNA-mouse-RT-qPCR	F: GAAGTTTTCTGCAAGTGGAGAG<br>R: CAGGCTCATTCATCTCTATGGT
MKI-mouse-RT-qPCR	F: CCTGGTCTTAGTTCCGTTGA<br>R: GTTGGCGTTTCTCCTCTTTTC
CDK4-mouse-RT-qPCR	F: GAGTGTGAGAGTTCCTAATGGA<br>R: GGTCCTGGTCTATATGCTCAAA
cyclin B-mouse-RT-qPCR	F: AACTTCAGCCTGGGTCG<br>R: CAGGGAGTCTTCACTGTAGGA
cyclin D-mouse-RT-qPCR	F: CGTATCTTACTTCAAGTGCGTG<br>R: ATGGTCTCCTTCATCTTAGAGG
cyclin E-mouse-RT-qPCR	F: CAGAGCAGCGAGCAGGAGC<br>R: GCAGCTGCTTCCACACCACT
mouse-*β-actin*	F: CCCATCTACGAGGGCTAT<br>R: TGTCACGCACGATTTCC
PCNA-sus-RT-qPCR	F: ATTTGGCCATGGGCGTGAAC<br>R: CTAGTGCCAAGGTGTCTGCAT
MKI-sus-RT-qPCR	F: ATTCCAGAAAGCACCAGGCT<br>R: TCCACTGTCTTCTCAGGGGT
CDK4-sus-RT-qPCR	F: GCCCTCAAGAGCGTAAGA<br>R: GCAGTGGCACAGACATCC
cyclin B-sus-RT-qPCR	F: AATCCCTTCTTGTGGTTA<br>R: CTTAGATGTGGCATACTTG
cyclin D-sus-RT-qPCR	F: TACACCGACAACTCCATCCG

	R: GAGGGCGGGTTGGAAATGAA
cyclin E-sus-RT-qPCR	F: CAGAGCAGCGAGCAGGAGC<br>R: GCAAGCTGCTTCCACACCACAT
sus-*β-actin*	F: CATCACCATCGGCAACGA<br>R: GCGTAGAGGTCCTTCCTGATGT
AK4-sus-RT-qPCR	F: GAACATCAAGGCCAACACGG<br>R: GGCGTGTGATCAAGTGGTCT
AK4-mouse-RT-qPCR	F: AGAACTTTGGCCTCCAGCAT<br>R: GTACTGTTTTGCCACGTCACC
U6	F: CTCGCTTCGGCAGCACA<br>R: AACGCTTCACGAATTTGCGT
URP	GTCGTATCCAGTGCAGGGTCCGAGGTATTCGCAC-TGGATACGACAGCTAT
miR-31-5p-RT	F: GCGAGGCAAGATGCTGGC<br>R: AGTGCAGGGTCCGAGGTATT

Table S1-B. Primers for vector construction

Names	Sequence (5′-3′)
Circ-06958-PCD2.1	F: GGGGTACCTGAAATATGC-TATCTTACAGAAAACATTCAGTCCTTCCTCCG<br>R: CGGGATCCTCAAGAAAAAATATATTCACCTG-TATTTTGTGGAAGATCAGTTTA
Circ-06958-WT-psiCHECK2	F: CCGCTCGAGAAAACATTCAGTCCTTCCTCCG<br>R: ATAAGAATGCGGCCGCCTGTATTTTGTGGAA-GATCAGTTTA
Circ-06958-MUT-miR-31	F: TACTCTGATCTCTTCTCGCCCATAT<br>R: ACATATGGGCGAGAAGAGATCAGAG
AK4-MUT-miR-31	F: AAGCATCATTGGCTCCTGCTGCAG<br>R: AGGAGCCAATGATGCTTCACGTTT
AK4-pCMV-HA	F: CGGAATTCCGATGGCTTCCAAACTCCTGC<br>R: GGGGTACCCTATGCCGCATCCTTGTACTG
AK4-WT-psiCECK2	F: CCGCTCGAGGAGTGTTCGGTTCCGTTTT<br>R: ATAAGAATGCGGCCGCGCTGTTCTTGATGTGAGGC

Table S1-C. Sequences of mimics and inhibitor of miR-31-5p, siRNAs against AK4

Names	Sequence (5′-3′)
miR-31-5p-mimics	AGGCAAGAUGCUGGCAUAGCU
miR-31-5p-inhibitor	AGCUAUGCCAGCAUCUUGCCU
mimics-NC	UCACAACCUCCUAGAAAGAGUAGA
Inhibitor-NC	UCUACUCUUUCUAGGAGGUUGUGA
AK4-siRNA-mouse-393	GGACAUUAGUACAGGCAGATT
	UCUGCCUGUACUAAUGUCCTT
AK4-siRNA-mouse-636	GGUACAAGGAUGCGGCAAATT
	UUUGCCGCAUCCUUGUACCTT
AK4-siRNA-mouse-705	GGACGGAGACUAACAGAAUTT
	AUUCUGUUAGUCUCCGUCCTT
AK4-siRNA-mouse-NC	UUCUCCGAACGUGUCACGUTT
	ACGUGACACGUUCGGAGAATT
AK4-siRNA-sus-692	CGACAAACCUGAAGCAGUUTT<br>AACUGCUUCAGGUUUGUCGTT
AK4-siRNA-sus-331	GGGAGAACAUCAAGGCCAATT
	UUGGCCUUGAUGUUCUCCCTT
AK4-siRNA-sus-430	UGUCGGAGCUGGAGAAUAGTT<br>CUAUUCUCCAGCUCCGACATT
AK4-siRNA-sus-NC	UUCUCCGAACGUGUCACGUTT
	ACGUGACACGUUCGGAGAATT
